# A Pilot Study of an Exercise-Based Patient Education Program in People with Multiple Sclerosis

**DOI:** 10.1155/2014/306878

**Published:** 2014-12-21

**Authors:** Stephanie Kersten, Mohammed Mahli, Julia Drosselmeyer, Christina Lutz, Magnus Liebherr, Patric Schubert, Christian T. Haas

**Affiliations:** ^1^Faculty of Health & Social Sciences, Institute for Complex Health Research, Hochschule Fresenius, University of Applied Sciences, Limburger Street 2, 65510 Idstein, Germany; ^2^BAG, Federal Institute for Human Posture and Movement Development, 65207 Wiesbaden, Germany; ^3^Hochschule Fresenius, University of Applied Sciences, 65510 Idstein, Germany; ^4^Sports Science Institute, Saarland University, 66123 Saarbrücken, Germany

## Abstract

There is increasing evidence that physical exercise leads to numerous positive effects in PwMS. However, long-term effects of exercise may only be achievable if training is implemented in daily routine. Enabling patients to exercise regularly, we developed a patient education program focused on evidence-based information of training. PwMS were educated in neurophysiological effects of physical exercise, exercise-induced benefits for PwMS, and risk factors (e.g., weather). Fifteen PwMS were analyzed before (*T*_0_) and after (*T*_1_) a 12-week patient education. Afterwards, participants performed their exercises autonomously for 32 weeks and were tested in sustainability tests (*T*_2_). Guided interviews were carried out, additionally. Significant improvements from *T*_0_ to *T*_1_ were found in 6MWT, gait velocity, TUG, fatigue, and quality of life. Significant results of TUG and gait velocity from *T*_1_ to *T*_2_ demonstrated that participants kept few effects after the 32-week training phase. Qualitative analyses showed improved self-confidence and identified training strategies and barriers. This pilot study provides evidence that PwMS are able to acquire good knowledge about physical exercise and apply this knowledge successfully in training management. One might conclude that this exercise-based patient education seems to be a feasible option to maintain or improve patients' integral constitution concerning physical and mental health.

## 1. Introduction

Multiple sclerosis (MS) is defined as a chronic inflammatory neurodegenerative disease of the central nervous system [1]. It is characterized by a large variety of symptoms, psychiatric problems, and motor control disorders [1, 2]. In Germany, about 120,000–140,000 people are affected by this disease, frequently diagnosed and manifested between the age of 20 and 40 [3]. Due to the unforeseen disease progress, in many cases the MS diagnosis means a huge psychological and physiological burden both upon the individual person, their careers, and family.

The pharmaceutical treatment still represents the key strategy in MS therapy. However, physical exercise is increasingly considered to be an important symptomatic and supportive intervention for PwMS in the therapeutic context. Numerous well-controlled studies were published examining the effects of physical exercise in PwMS and a variety of different exercise interventions have been tried (aerobic training, resistance training, combined programs, etc.). In general, there is strong evidence that PwMS who do exercise regularly perform better in terms of muscle power function, exercise tolerance functions, and mobility-related activities compared to patients who do not [1, 4, 5]. Furthermore, physical exercise seems to be a feasible option to improve not only in aerobic capacity and muscle strength, but also in fatigue, gait, balance, and quality of life [6–17]. Both endurance and resistance training are well tolerated by PwMS [18]. Additionally, endurance training seems to have a possible beneficial effect beneath physiological factors on the psychological profile of PwMS [1, 7, 9, 19, 20].

With respect to these positive findings, one might expect that PwMS generally know physical exercise as a therapy option, perform exercises autonomously, and participate in exercise therapy interventions. In contrast, there are indications that PwMS show reduced physical activity, a nescience and uncertainty about physical exercise in MS disease—possibly due to missing exercise recommendations [7, 21, 22]. As a consequence, there is an increasing risk in developing secondary diseases. Therefore, PwMS suffer more often from obesity, diabetes mellitus, osteoporosis, and cardiovascular diseases [7, 21, 23]. Further investigations in animal models show that decreasing mobility may accelerate neurodegenerative processes, in both neurotraumatic disorders like spinal cord injury and neurodegenerative diseases like multiple sclerosis and Parkinson's disease [24–31].

Enabling PwMS to implement regular physical exercise in their everyday life, we developed an exercise-based patient education program. Because of the individual character and adaptation processes of exercise-induced effects per se, there is a strong need for an autonomous performance of physical exercise especially in persons with neurological disorders. It is highly important that the patient education program relies on strong evidence-based information about the effects, interactions, and risks of physical exercise in MS disease. Until now, patient education is established especially in chronic diseases like diabetes mellitus, rheumatism, chronic obstructive pulmonary disease (COPD), and asthma [32–35]. Regularly offered exercise groups, mostly cofinanced by health insurances, have already been integrated in the long-term rehabilitation for persons with heart diseases (e.g., after heart attack) and lung diseases. But standardized therapeutic education programs for PwMS are rare. Furthermore, existing self-care programs focus primarily on symptom and disease management (e.g., pain, fatigue), not on exercise recommendations and behavioral interventions to increase physical activity [36]. The development and evaluation of an exercise-based patient education for PwMS is more problematic and complicated due to many factors: symptom variety, different disability status, conflicting interaction with physical effort needed for activities of daily living (e.g., house cleaning, cooking), local dependency of patients, and methodical approaches for empirical examinations. Therefore, only few previous studies focusing on concepts of exercise-based patient education or therapeutic education have been published. Considering these present studies, predominantly internet-based interventions have been conducted. The research team around Dlugonski et al. [37] and Motl et al. [38] see a possible key strategy in increasing PwMS' physical activity using internet intervention for a long-term behavioral change. The results confirmed the efficacy of internet intervention for increasing physical activity in persons with MS in both objective and self-report measures [37, 38]. A German research group also developed an internet intervention to support PwMS in home-based physical exercise [39, 40]. The authors conducted an introduction seminar and a following internet-based e-training providing an online community to interact with others or a therapist in order to modify physical activity in daily living. Gutiérrez et al. [41] examined the usefulness and effectiveness of a telerehabilitation program for PwMS using virtual reality-video games. The authors concluded that those virtual reality-video games might be a possible training therapeutic alternative if conventional treatment is not available [41].

Although first positive effects of the internet-based interventions have been shown, we miss two main key aspects in previous exercise-based patient education programs with PwMS: (1) via internet a lot of people can be reached, but provided exercises are very unspecific and (2) not every patient is comfortable with the new media. Therefore, the objective of the present study was to evaluate an exercise-based patient education program in order to teach patients' basic training principles, skills in training adaptations, and home-based exercise. It is important for PwMS to learn more about the interaction of physical exercise with MS-related symptoms in everyday life and the rest between sets of different exercises, and to be aware of possible contraindications to exercise, knock-out criteria such as heat, cold, and fatigue. In contrast to previous interventions, in every session of this program theoretical and practical contents have been combined. It was hypothesized that PwMS transfer their training experiences and knowledge into their daily routine and manage a workout successfully and independently beyond the intervention.

## 2. Materials and Methods

### 2.1. Patients

The pilot study was conducted based on a cooperation of the German MS Society (Department of Saarland), the Sports Science Institute of Saarland University (Saarbrücken), and the Hochschule Fresenius, University of Applied Sciences (Idstein). Study participants were recruited within an information event in March 2010 at the Saarland University, publicized and promoted through the press office of the Saarland University as well as the German MS Society specifically within the German province Saarland via Internet and daily press. All interested persons were invited to this information event where project procedure, study background, and objectives were explained, inclusion and exclusion criteria were described, questions were answered, and interested patients were able to sign in for possible participation. The inclusion criteria were as follows: (a) patients were over 18 years old, (b) had a confirmed diagnosis of MS, (c) passed a medical checkup prior to baseline ensuring they were free of any acute cardiovascular disease, (d) were impaired because of disease-related symptoms, (e) had certain standing and ambulatory abilities, (f) did not receive other active therapeutic treatments during the 12-week patient education program, and (g) were able to participate in all tests and the patient education program (three sessions/week of 60–90 min/session, maximal three missing sessions allowed per participant) at the Sports Science Institute in Saarbücken. Exclusion criteria were as follows: (a) patients were diagnosed with another chronic disease, (b) had a relapse within one month prior to baseline, (c) failed the medical checkup, (d) changed the medication prior to baseline, and (e) did not absolve the tests and the 12-week patient education program. Out of the 21 registered PwMS, 19 were recruited for the experimental group, because two patients failed the medical checkup prior to baseline tests. The degree of neurological impairment in MS has been quantified by the Expanded Disability Status Scale (EDSS) [42] and the Neurologic Rating Scale (NRS) [43]. Each participant gave informed consent prior to the study. An overview of participant data concerning actual clinical course, medication, or therapy interventions prior to baseline tests (sports, physiotherapy/occupational therapy) is shown in Table 1.

### 2.2. Intervention

The theoretical concept was embedded in applied exercise sessions which aimed at educating patients in training principles, removing patients' possible fear about physical exercise, increasing patients' empowerment, and enabling patients to exercise beyond the project. The intervention was divided in two phases: an instructed training phase (six weeks) and an assistive training phase (six weeks). The first phase consisted of a theoretical and practical education program containing coordination (e.g., highly reflex-based movements, balance training, active games), endurance (e.g., dancing, aerobics, walking on different surfaces like in the forest or at sand), and strength training (e.g., device-independent body weight training, elastic band). With respect to research findings, coordination, aerobic, and progressive resistance training in PwMS are well tolerated and show positive effects in patients' performance [5, 7, 9, 10]. Additionally, evidence-based neurophysiologic effects were explained to understand exercise-induced effects in humans and interactions with MS disease. In particular, theoretical contents in our patient education program concerning physical exercise and neurophysiology are based on research literature [5, 7, 27, 28]. Representative results have been published in 2004 in the Journal* Brain*, in which meaning and efficacy of highly reflex-based movement with respect to generated neurophysiologic effects were accentuated [27].

**Figure 1:**
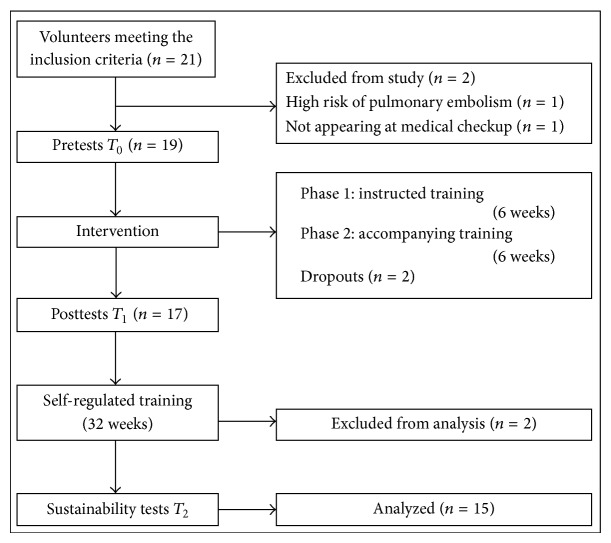
Study design.

Patients were encouraged to find their individual training limits, weaknesses, and exercise goals and to learn more about their individual exercise knock-out criteria (e.g., hot temperature) and the avoidance of negative interactions with exercise behavior in daily routine (e.g., being rested for sports, meaning not to do the house cleaning on a training day). In the assistive training phase, patients planned and realized their exercise program more autonomously. Former training tutors now changed their role into assistants, helping and supporting participants in unclear or dangerous situations. Within this transition phase, patients should become more independent in performing their exercises and they were free to decide whether training sequences were absolved at home or at the university. After the 12-week patient education program, PwMS performed their exercises autonomously for 32 weeks. The complete study design is shown in Figure 1. Structure and program contents are shown in Table 2.

Three working hypotheses for further quantitative and qualitative analyses were formulated.

(H_1_) A 12-week exercise-based patient education program leads to significant improvements in physical working capacity, health-related quality of life, fatigue, and self-efficacy in sportive activities in PwMS.
(H_2_) Patients maintain the predicted results in *T*_2_ and remain significantly* constant* in comparison to the results of *T*_1_ in physical working capacity, health-related quality of life, fatigue, and self-efficacy in sportive activities.
(H_3_) A 12-week exercise-based patient education program improves the empowerment of PwMS and leads to a change in individual training and therapy management beyond the intervention.

### 2.3. Outcomes

Subjects were tested at three test times (*T*_0_, *T*_1_, *T*_2_) in endurance capacity, mobility, fatigue, self-efficacy in sportive activities, and health-related quality of life (HrQoL). Guided interviews were carried out, additionally. The endurance capacity was measured by the Six-Minute-Walk-Test (6MWT) [44] and physical working capacity by treadmill analysis. The treadmill test began at 2.0 km/h, velocity increased every two minutes by 0.3 km/h. Breakup criteria were the subjects' maximal point of exhaustion and maximum speed level, the maximum speed level of 5.0 km/h, subjects' increasing fear of falling, or the project leader stopping the test for safety reasons (subject stumbled or slipped). The maximum speed level (km/h) was accepted when the whole speed level was held for at least one minute. Mobility was tested with the Timed-Up-and-Go-Test (TUG) [45]. The time in seconds (s) for a distance of twice three meters was measured, starting with the command “Go” and ending when the patient sat down and leaned back. The TUG was conducted three times with a self-chosen break between each set and the fastest trial was used for further analyses. Fatigue was quantified with the Fatigue-Severity-Scale (FSS) [46]. Self-efficacy was measured with the German Self-efficacy-of-Sportive-Activities (SSA) questionnaire [47]. HrQoL was analyzed with the SF-36 [48]. Subjects were requested to appear rested for each test.

### 2.4. Quantitative Data Analyses

Power calculation to determine sample size was not taken into account due to the fact that the number of participants in the treatment group was established by the number of patients volunteered for the project. Normal distributions of the data were analyzed. Descriptive statistics are presented in text and tables as mean ± standard deviation (M ± SD). Depending on the distribution of the data, paired-sample *t*-tests (normal distribution assumed) for within group changes or Wilcoxon signed rank test (normal distribution rejected) was used. Concerning the first hypothesis that PwMS significantly improve their physical working capacity, HrQoL, fatigue, and self-efficacy in sportive activities after the 12-week intervention, the study variables from *T*_0_ to *T*_1_ within the exercising group were compared using a paired, one-sided *t*-test (for ordinal scaled data: Wilcoxon signed rank test). Statistical *α*-error was set at *α* ≤ 0.05. Effect sizes based on differences in mean scores were expressed as Cohen's *d*.

Focusing on follow-up effects, we secondly hypothesized that the results of *T*_2_ remained significantly* constant* in comparison to the results of *T*_1_. Instead of the calculation of the *P* value (*α*-error), which is the adequate procedure for analyzing the statistical differences, the statistical *β*-error was used in order to analyze a significant nondifference in the hypothesis [49]. “Real” hybrid significance test was applied for proving the second hypothesis [49, p. 82], [50, p. 120]. The *β*-error estimates the probability to accept the null hypothesis (equivalent mean values) under the condition that the alternative hypothesis is true. A 10% difference of the test score *T*_1_ compared to the score of *T*_2_ was considered as constancy. Therefore, the fictitious sample was composed of each subject's score at *T*_1_ minus 10%. A significant upper deviation (acceptance of the alternative hypothesis) of this 10% was rated as inconstancy. If the difference between *T*_1_ and *T*_2_ did not significantly decrease by more than 10% of *T*_1_, the effects of the patient education program were considered to have been maintained [50]. If there had been significant improvements in study variables from *T*_0_ to *T*_1_ we next calculated the *β*-error of a one-sided, paired *t*-test (for ordinal scaled data: *β*-error of Wilcoxon test). The statistical *β*-error was set at *β* ≤ 0.05 and compared to the *P* value (*β*-error). According to Bortz [50], the setting of the decrease considered as nonconstancy (here 10%) must be extensively justified as there are no existing conventions. The decision for a 10% level was set as a reasonable minimum limit in order to refer to constant results. For an overview of the whole study duration, a paired one-sided *t*-test (for ordinal scaled data: Wilcoxon signed rank test) was performed to compare the study results from *T*_0_ to *T*_2_, whereby the interpretation of these results was certainly limited. All statistical analyzes were conducted using SPSS.

### 2.5. Qualitative Data Analyses

The third working hypothesis was proved via qualitative analyses. Within the context of the overall survey, three guided interviews per participant were conducted at predefined points in time: guided opening interview (*T*_0_) before starting the project, guided final interview (*T*_1_) after finishing the 12-week patient education program, and all psychometric and physiological posttests and guided sustainability interview (*T*_2_) after the 32-week training phase. All guided interviews were carried out by one person, without observer according to the recommendations of Kuckartz [51]. All interviewees were informed about content and objectives of the interviews before starting. The interview was carried out and structured by a guided interview paper. At the end of each interview, the interviewer was encouraged to check all open-end questions on the interview paper with respect to avoiding forgotten important contents.

The interviewer was encouraged to arrange a friendly atmosphere and was free to enquire, especially in situations where the interviewee stagnated or was not sure to proceed. Interviewees were coincidental characterised and named via the numbers given prior to all tests in *T*_0_. During the interview situation of approximately 30 minutes per interviewee, telephones as well as mobile phones were switched off and a “Please don't disturb” sign at the door of the interview room was posted. The interviews were recorded directly via dictation machine. After each interview, the qualitative data were directly marked with the participant's number and point in time and saved on  .mp3 files on external hard disks by the interviewer. Considering the transcription guidelines of Kuckartz [51], all interviews were transcribed word for word via transcription software. The transcribed interviews were analyzed by a computer-assisted software MAXQDA^+^ (Version 10). The primary inductive formed categories (and subcategories) are shown in Table 3.

Considering the primary unknown phenomena due to a patient education's success in sustainable home-based physical exercise of the participants, the inductive content analysis was used. Content analysis is a systematic and objective research method to quantify phenomena. Two independent reviewers translated the transcribed data into codes and categoized all coding segments. Both reviewers interpreted the statements of the categories independently at first. In a final discussion both reviewers achieved a consensus with regard to varying categorizations.

## 3. Results

The whole trial took place from April 2010 to April 2011 at the Sports Science Institute at Saarland University in Saarbrücken, Germany. Fifteen PwMS (men = 3, women = 12) on average 48.1 ± 9.2 years old (men = 45 ± 12.1 years, women = 48.8 ± 8.9 years) with a mean disease duration of 10.9 ± 7.7 years from the point of diagnosis, a current EDSS score of 4 ± 1.5, and NRS score of 78.2 ± 10.2 completed the exercise-based patient education and were included in further statistical analyses (Table 4). Proband number 2 cancelled the participation during the program because of an orthopedic diagnosis (spondylolisthesis). Number 3 broke off for personal reasons. Number 9 must be considered as a single case, because he differed a lot compared to all other participants concerning his disability status. Number 21 received chemotherapy in the end of the patient education program. The data of all 4 dropouts have been excluded for further statistical analyses.

### 3.1. Results of Quantitative Analyses

Table 5 provides all statistical analyses of the interval scaled data. Apart from the self-efficacy of sportive activities (SSA) and the physical functioning (SF-36), all variables showed significant improvements after the 12-week intervention.

Participants showed highly significant improvements with a high effect of the intervention in the TUG-Test. Analyses indicated that the participants could keep these effects significantly within the self-regulated training phase. These tendencies were confirmed by the comparison from *T*_0_ to *T*_2_. Data of the 6MWT revealed a highly significant improvement from *T*_0_ to *T*_1_ accompanied by a high effect. Further analyses showed that these positive effects did not remain in *T*_2_. Treadmill tests showed a significant improvement in maximum time and speed after the intervention with high effect sizes. Further analyses showed that patients significantly kept these effects. This can be confirmed with *t*-test analyses from *T*_0_ to *T*_2_. Fatigue significantly decreased subsequently to intervention with a moderate to high effect. These effects have not been maintained after the 32-week self-regulated training phase. Three dimensions of the SF-36, general health, vitality, and mental health, showed significant improvements from *T*_0_ to *T*_1_ with moderate to high effects. Although none of these dimensions remained significantly constant after the 32-week training phase, vitality indicated a significant improvement over the whole study period.

Only one dimension of the SF-36, bodily pain, showed at least a significant improvement after the 12-week intervention (Table 6). This effect could not be kept up by PwMS after the self-regulated training phase 32 weeks later.

### 3.2. Results of Qualitative Analyses

In Table 7 the results of the qualitative analyses using the computer-assisted software are demonstrated. In total, 1934 coded segments were assigned to the categories. Additionally, brief summarizes of the qualitative results are demonstrated (Table 7).

Representative quotations have been chosen to support the interpretation. The motivation strategy and the training organization seemed to be interrelated: intrinsic motivated patients showed a more regular and specific exercise whereas extrinsically motivated PwMS exercised more irregularly, for example, since they missed the training group: “*That's why it is better, that I am doing my exercises in a group and have an appointment outside the home.*” Others were happy about the possibility to exercise independently at home knowing the benefits in terms of the disease: “*In this project, it was directly used and trained on MS specific needs (…) so that I benefited immensely (…) and benefited in that way, that I can direct my own training at home.*” Most patients placed training and rest periods in their daily routine, meaningfully: “*Rest periods, they are better-placed by me (…) I am not that exhausted the whole day.*” Different strategies of implementing training in everyday life could be uncovered: “*I can better discipline myself (…) I was thinking about set hours (for training) depending on how it fits in my life.*” Independency seemed to be a major factor to enable regular physical exercise: “*I had my elastic band with me in our holiday and used it for training.*” Training barriers and knock-out criteria have been identified which prevented participants from exercise: “*(…) if you come home in the afternoon at 4 pm, you are knocked-out (…) I can't do my exercises anymore.*”

Although all participants showed a well-managed training behavior, only half of them were able to explain the theoretical basis: “*Aerobic exercises are a constant movement in order to work out over a longer period. (…) for me cycling is endurance training.*” Interviews showed an improved self-confidence and patients performed better in activities of daily living: “*Blow-drying my hair for instance (…) brushing teeth (…) or I worked with a hammer (…) that all ameliorated, too (…)*”* and* “*In my household, I am so much more active than before.*” The subjective attitude towards physical exercise and training was totally positive. Tests and theoretical and practical sessions have been well tolerated by all patients. Every participant was convinced of the positive exercise-induced effect and the well-being resulting after a training session: “*What I already mentioned and found positive within this project: that I dared to do many things. Inside of this MS training group and with this support, that was so important in order to improve the self-confidence. If you are handicapped, you wouldn't rely on yourself even though you are able to do many things you wouldn't expect.*”

## 4. Discussion

The aim of the present study was to conduct an exercise-based patient education program in order to ensure participants training management beyond the project. Previous authors have studied the effects of Internet-based interventions or telerehabilitation programs regarding exercise-based patient education [37–41]. According to literature, the potential of exercise as a therapy option in neurological diseases is high [4, 9, 28]; however the interaction of exercise and MS disease involves not only chances but also risks. To our knowledge, this publication is the first to evaluate an exercise-based patient education containing theoretical and practical aspects in a face-to-face group training form.

The quantitative results of our study demonstrate improvements in PwMS' mobility, gait ability, endurance, fatigue, and health-related quality of life after completing the 12-week intervention. Numerous previous studies confirm the positive effects of regular physical exercise in PwMS concerning aerobic capacity [6, 52], strength [10, 13, 20, 53], coordination and walking ability, respectively [20, 52], and quality of life [11, 54–56]. Tallner et al. [57] outlined in their examination using a self-report questionnaire that the level of physical exercise is not associated with clinical disease activity or relapse occurrence. Considering the results of the SSA questionnaire, no improvements could be found in our examination. One reason for these results might be the MS-unspecific character of the SSA questionnaire. However, previous studies demonstrated positive effects of exercise in PwMS' self-efficacy [55, 58].

Summarizing the results in physical working capacity and quality of life, numerous well-controlled studies demonstrate homogenous results. Regarding fatigue, heterogeneous results can be found [11, 12, 52, 56, 59, 60]. While Dalgas et al. [11] reported significant improvements in fatigue after a strength training period, van den Berg et al. [52] found no effect of endurance training in fatigue. Consulting additional studies, Andreasen et al. [12] concluded that exercise therapy might have the potential to induce positive effects in PwMS' fatigue. In this context, Sabapathy et al. [18] examined the type of sports concerning the fatigue effects in MS. The authors concluded that on the one hand endurance and resistance training are well tolerated by PwMS [18]. But on the other hand they could not differentiate fatigue effects in consequence of both types of exercises [18].

Considering previous publications with regard to follow-up measurements and/or sustainability tests, underlying results are presented scarcely and unclear. Dalgas and colleagues [10] showed that the improvements of a 12-week resistance training with PwMS persisted at a 12-week follow-up conducting self-guided physical activity. Besides, van den Berg et al. [52] conducted a 4-week aerobic treadmill training which was feasible and well tolerated by participants. The results after a training period indicated significant differences in walking endurance and after further 4 weeks of a rest period the results returned to baseline. Both studies did not involve a patient education strategy and however conducted a supervised training program with PwMS within a fixed period.

The concept of patient education is established especially in diabetes mellitus, rheumatism, chronic obstructive pulmonary disease (COPD), and asthma [32–35]. There are many positive effects of patient education in different chronic diseases, but programs for PwMS are very rare and they focus primarily on pain, fatigue, cognition, or general health [36]. Shevil and Finlayson [61] conducted a 5-week cognitive self-management program with PwMS. Disease knowledge and management increased by over one-half of the participants by approximately 50% and improvements in self-efficacy were measured by the Cognitive Management Self-efficacy questionnaire [61]. Further positive effects on PwMS' self-management and self-efficacy after a disease self-management course were presented by Barlow and colleagues [62]. Patients' physical and psychological impairments reduced significantly after the intervention [62]. Patient education focused on cognitive interventions, disease-management, and coping strategies, but there is a lack of competence transfer considering physical exercise in MS disease. Future perspectives rely on the development of patient education programs transferring besides psychological management strategies evidence-based physical exercise possibilities.

## 5. Conclusions

The partial success of the current exercise-based patient education is represented partly in quantitative data analyses. Therefore, influencing factors like training management motivation, knowledge, and changes in training skills are evaluated in qualitative analyses. Considering the qualitative results of our study, PwMS were able to acquire good knowledge on basic physiological functions and training principles and succeeded in applying this knowledge to their training management. Changes in patients' therapy and training empowerment were illustrated through interview statements. The transfer of competences (self-competence, empowerment, etc.) are preconditions not only for realizing a training program effectively but also to coordinate training and everyday challenges and finally to assure sustainable effects. Nevertheless, more research work is required to modify this patient education program with regard to the different patient profiles, motivation, and coping strategies. The following suggestions for improvements have been stated by participants: (1) shortening the patient education program (e.g., six weeks in total, twice a week, a mix of instructed and assisted phases), (2) implementing regular meetings with those who cannot motivate themselves for external motivation, (3) developing of teaching material, for example, booklets, DVDs, and (4) offering a refresher course (e.g., once or twice a year) to keep participants up to date in current research work and repeat theoretical and practical aspects of physical exercise. Thus, it can be observed that the educative part of this program needs to be repeated in certain time intervals or within the use of other media modules to achieve a higher level of sustainability. 

*Study Limitations.* Because of the difficult methodological approach, this examination underlies several limitations. The main limitation is the lack of a control group which limits the definite conclusions. Due to the explorative character using an exercise-based patient education program in PwMS, this pilot study was conducted in a quasi-experimental design. In further research, experimental designs are required. A nonprobability sampling was used for participant selection. Only inclusion and exclusion criteria were set for recruited patients. Immeasurable factors such as training sessions, self-chosen exercise determinants, disease progress, motivation for physical exercise, and patients' different characteristics may confound the results over a study period of one year. The method of recruitment may have led to selection bias, as PwMS who participate in a patient education program learning about physical exercise often show a certain motivation for health-promoting exercise behaviors. The measurement of strength parameters were missing in this study and should be considered for future trials. Considering the statistical analyses, the decision of a 10% decrease of the test score at *T*_2_ from the mean value at *T*_1_ as the stability margin was made due to the estimation by practical and therapeutical experiences based on a subjective definition. In total, the intervention duration of 12 weeks should be shortened in following patient education programs due to the accessibility for PwMS. In this context, further education designs must be developed to enable more PwMS, especially working persons, to participate in similar exercise-based patient education programs. Weekend workshops would be conceivable; they however have to be evaluated with an adequate and systematic methodological design.

## Figures and Tables

**Table 1:** Overview of data of the 19 participants at the beginning (April 2010).

Prob. number	Gender (*m* = 1, *f* = 2)	Age at study beginning	Diagnosis (year)	Actual clinical course	Medication	EDSS	Scripps	Sports/physiotherapy/occupational therapy
2	2	67	1994	Secondary progredient	No basic therapy	6.5	52	2x weekly
3	2	46	2001	Secondary progredient	Betaferone	6	48	2x weekly
4	1	34	2003	Relapse-remitting	Tysabri	3	79	None
5	2	56	1999	Primary progredient	Only homeopathy	5	61	Yes
6	2	41	2004	Relapse-remitting	Copaxone	3.5	74	1-2x weekly
7	2	56	1998	Relapse-remitting	Avonex	4.5	87	None
9	1	59	1996	Secondary progredient	No basic therapy	6.5	64+	2x weekly
10	2	44	2007	Relapse-remitting	Betaferone	3.5	73	Rehability sports
11	2	38	1997	Relapse-remitting	Copaxone	2	93	None
12	2	60	1983	Secondary progredient	None	6.5	74	Yes
13	2	46	2002	Secondary progredient	L-Thyroxine	3.5	84	Yes
14	1	58	1996	Secondary progredient	Interferone	6	78	Yes
15	1	43	2007	Primary progredient	No basic therapy	2	94	None
16	2	57	1995	Primary progredient	No basic therapy	4.5	73	2x weekly
17	2	49	1983	Relapse-remitting	Betaferone	6.5	67	2x weekly
18	2	45	2001	Secondary progredient	Copaxone	4	68	Yes
19	2	34	2004	Relapse-remitting	Only homeopathy	2	94	None
20	2	60	2008	Relapse-remitting	No basic therapy	3.5	74	Yes
21	2	49	1993	Secondary progredient	Mitoxantrone	4.5	57	2x weekly

**Table 2:** Contents of the exercise-based patient education (in chronological order).

Week	Training phase	Objectives	Theoretical contents	Practical contents
1-2	Instructed	Coordination training	Reflex-based activities Neurophysiologic principles	Deviance-based gait training Balance training Active games

3-4	Instructed	Endurance training	Cardiovascular basics Exercise determinants Training principles	Aerobic (Nordic) walking Dancing

5-6	Instructed	Strength training	Neuromuscular basics Exercise determinants Muscle conditioning	Device-independent strength training with own body weight Training with elastic band

7-8	Assisted	Individual combined training	Training management/considering environmental conditions	Self-regulated exercise and rest periods Feedback from assistants

9-10	Assisted	Individual combined training	Training management/considering environmental conditions	Self-regulated exercise and rest periods Feedback from assistants

11-12	Assisted	Individual combined training	Training management/considering environmental conditions	Self-regulated exercise and rest periods Feedback from assistants

**Table 3:** Categories and subcategories for qualitative analyses.

Category	Code	Subcategories
Complex (I)	Motivation	Intrinsic motivation Extrinsic motivation

Complex (II)	Training & therapeutic management	Training management before project Management with rest periods before project Therapeutic treatment before project Regular physical exercise before project Management with rest periods after project Therapeutic treatment after project Exercise behavior in daily living Regular physical exercise after project

Complex (III)	Training barriers	

Complex (IV)	Knowledge interrogation regarding training theory	

Complex (V)	Knock-out criteria	

Complex (VI)	Quality of Life	Perceived alterations Alterations in motor control Feedback from social environment Activities in daily living Fatigue/fatigue management

Complex (VII)	Subjective attitude towards physical exercise	

Complex (VIII)	Criticism and tips for further patient education programs	

**Table 4:** Characteristics of the experimental group.

Variables	Exercise group
Sex	
Male [*n*]	3
Female [*n*]	12
Age in years [M ± SD]	48.1 ± 9.2
Years since diagnosis [M ± SD]	10.9 ± 7.7
MS subtype	
Relapse-remitting [*n*]	8
Secondary progressive [*n*]	4
Primary progressive [*n*]	3
EDSS [M ± SD]	4 ± 1.5
Scripps [M ± SD]	78.2 ± 10.2

**Table 5:** M ± SD, reported for *T*_0_, *T*_1_, *T*_2_, within exercising group comparison from *T*_0_ to *T*_1_ using *P* values and effect sizes (*α*, paired one-sided *t*-test, *P* ≤ 0.05); if significant *P* value was reported from *T*_0_ to *T*_1_, significance tests were calculated for *T*_1_ minus 10% and *T*_2_ (*β*, paired one-sided *t*-test, *P* ≤ 0.05); additionally, *T*_0_ to *T*_2_ comparisons were calculated using *P* values and effect sizes (*α*, paired one-sided *t*-test, *P* ≤ 0.05).

Outcome measure	*T*_0_ baseline	*T*_1_	*T*_2_	*T*_0_–*T*_1_ (*α*) *P* value	*T*_0_–*T*_1_ effect size *d*	*T*_1_–*T*_2_ (*β*) *P* value	*T*_0_–*T*_2_ (*α*) *P* value	*T*_0_–*T*_2_ effect size *d*
TUG [s]	9.8 ± 2.7	7.5 ± 2.3	8.1 ± 1.9	<0.001	3.38	<0.05	<0.001	2.6
6MWT [m]	419.2 ± 126.3	483.7 ± 140.2	432.9 ± 123.3	<0.001	−3.3	ns	ns	−0.73
Treadmill [min]	12.1 ± 5.5	15 ± 5	14.6 ± 5.5	<0.001	−1.91	<0.01	<0.05	−1.12
Treadmill [km/h]	3.5 ± 0.8	4 ± 0.7	4 ± 0.9	<0.001	−2.39	<0.001	<0.05	−1.23
Fatigue [score]	5 ± 1.6	4.5 ± 1.7	4.7 ± 1.5	<0.05	0.86	ns	ns	0.14
SSA [score]	4.9 ± 1.4	5.4 ± 0.8	5.2 ± 0.7	ns	−0.6	—	ns	−0.5
SF-36 [score]								
General health	58.9 ± 18.6	66.7 ± 18.1	61.3 ± 19.6	<0.01	−1.49	ns	ns	−0.15
Physical functioning	51.7 ± 19.3	56.3 ± 25.7	51.9 ± 25	ns	−0.57	—	ns	0.15
Vitality	44.3 ± 19.6	55 ± 18.6	52.7 ± 16.2	<0.001	−2	ns	=0.05	−0.91
Mental health	73.3 ± 13.2	77.3 ± 14.6	73.5 ± 13.1	<0.05	−0.97	ns	ns	0.22

**Table 6:** M ± SD, reported for *T*_0_, *T*_1_, *T*_2_, within exercising group comparison from *T*_0_ to *T*_1_ using *P* values (*α*, Wilcoxon test, *P* ≤ 0.05); if significant *P* value was reported for *T*_0_ to *T*_1_, significance tests were calculated for *T*_1_ minus 10% *T*_2_ (*β*, paired Wilcoxon test, *P* ≤ 0.05); additionally, *T*_0_ to *T*_2_ comparisons were calculated using *P* values (*α*, paired Wilcoxon test, *P* ≤ 0.05).

Outcome measure	*T*_0_ baseline	*T*_1_	*T*_2_	*T*_0_–*T*_1_ (*α*, Wilcoxon) *P* value	*T*_1_–*T*_2_ (*β*, Wilcoxon) *P* value	*T*_0_–*T*_2_ (*α*, Wilcoxon) *P* value
SF-36 [score]						
Bodily pain	83.9 ± 21.5	94.9 ± 9.1	86.1 ± 19.9	<0.05	ns	ns
Physical role	46.7 ± 46.1	63.3 ± 42.1	40.4 ± 40.2	ns	—	ns
Emotional role	73.3 ± 38.2	91.1 ± 26.6	79.5 ± 37.4	ns	—	ns
Social functioning	76.7 ± 22.6	82.5 ± 19.4	82.7 ± 16.6	ns	—	ns

**Table 7:** Results of coding.

Codes	Number of coded segments [*n*]	[%]	Results
Motivation	116	6	Training performance and implementation depended on motivation

Training & therapeutic management	435	22.5	Modified exercise behavior in daily living, meaningful implementation of exercise sessions and rest periods

Training barriers	91	4.7	Weather, fear of mistakes, social events, and missing motivation

Knowledge interrogation regarding training theory	153	7.9	Although all participants showed a well-managed training behavior, only half of participants were able to explain the theoretical basis

Knock-out criteria	39	2	Identification of individual knock-out criteria (e.g., fatigue, hot temperature, and high stress level)

Quality of life	247	12.8	Improvements in psychological and physiological parameters, especially in activities of daily living

Subjective attitude towards physical exercise	102	5.3	Unanimous positive

Criticism and tips for further patient education programs	109	5.6	Some participants postulated a training manual, a training DVD, regular meetings with the training group, and refresher courses

Others	642	33.2	Personal data, individual expectations, individual open questions, and other topics

Total codes	**1934**	**100**	
